# Immune Modulation in Cancer: The Role of Tumor‐Treating Fields (TTFields)

**DOI:** 10.1002/cpz1.70270

**Published:** 2025-12-05

**Authors:** Menglan Liu, Maria El Hage, Jihui Zhao, Michael Linnebacher

**Affiliations:** ^1^ Molecular Oncology and Immunotherapy, Clinic of General Surgery University Medical Center Rostock Rostock Germany

**Keywords:** cancer, immunotherapy, immunogenic cell death, TTFields

## Abstract

Tumor‐treating fields (TTFields) represent an innovative approach to cancer treatment that leverages low‐intensity (1‐3 V/cm) alternating electric fields operating at intermediate frequencies (100‐300 kHz). These electric fields are specifically designed to disrupt the mitotic process, thereby inhibiting the proliferation of malignant cells. Recent research has shown that TTFields not only induce direct cytotoxic effects but also modulate the immune system by triggering immunogenic cancer cell death, thereby enhancing immune cell infiltration into tumor sites. This dual mechanism opens up new possibilities for synergistic integration with immunotherapy, which has already revolutionized oncology through the advent of immune‐checkpoint inhibitors, adoptive cell therapies, and cancer vaccines. Given the immune‐modulatory properties of TTFields, there is growing interest in exploring their potential synergistic effect to enhance immunotherapeutic efficacy. Recognizing the complementary role of TTFields in cancer treatment paves the way for innovative combinatorial strategies that may further improve patient outcomes by enhancing the effectiveness of both TTFields and immunotherapy. © 2025 The Author(s). Current Protocols published by Wiley Periodicals LLC.

## Introduction

Cancer remains one of the greatest global threats to human health. Although traditional treatment modalities such as surgical resection, chemotherapy, and radiotherapy are widely used, they are often associated with limited specificity, serious complications, and a high risk of relapse or resistance. Therefore, an urgent need for innovative, effective, and less invasive therapeutic strategies remains.

In recent years, immunotherapy has emerged as the fourth pillar of cancer treatment (Waldman et al., 2020). Several approaches, including immune‐checkpoint inhibitors (ICIs), adoptive cell transfer, and cancer vaccines, have achieved promising results (Rui et al., 2023). These therapies aim to restore or enhance the body's natural immune surveillance mechanisms, enabling the recognition and elimination of malignant cells. Despite these advancements, challenges such as heterogeneous tumor immunogenicity, immune suppression, resistance to ICIs, and adverse side effects remain significant barriers to the broader efficacy of immunotherapy (Vinay et al., 2015; Kulkarni et al., 2025).

Tumor‐treating fields (TTFields) are a non‐invasive therapeutic modality that employs low‐intensity (1‐ to 3‐V/cm), intermediate‐frequency (100‐ to 300‐kHz) alternating electric fields to interfere with mitosis, thereby inhibiting tumor growth and inducing apoptosis (Kirson et al., 2004). TTFields have demonstrated clinical benefit in glioblastoma (GBM) and malignant pleural mesothelioma (MPM), resulting in their approval as a treatment approach by the U.S. Food and Drug Administration (Ceresoli et al., 2019; Stupp et al., 2015). Increasing evidence also supports their efficacy as an active antimitotic therapy in other solid tumors, such as non‐small‐cell lung cancer (NSCLC), pancreatic cancer, and ovarian cancer (Leal et al., 2023; Rivera et al., 2019; Vergote et al., 2018).

Beyond their cytotoxic effects, emerging evidence indicates that TTFields exert profound immunomodulatory effects. By inducing immunogenic cell death (ICD), releasing damage‐associated molecular patterns (DAMPs), and remodeling the tumor microenvironment, TTFields can enhance immune cell activation and infiltration (Voloshin et al., 2020). These immune‐modulatory properties are particularly relevant in the context of immunotherapy, in which resistance to therapy often arises from inadequate immune priming or suppression within the tumor microenvironment (Kawashima & Togashi, 2023). Thus, TTFields function not only as an antimitotic therapy but also as a potential sensitizer to immune‐based approaches.

More importantly, a post‐marketing surveillance study encompassing more than 25,000 patients confirmed that TTFields have an exceptional safety and tolerability profile, with no systemic or organ‐specific toxicities identified. Adverse events are predominantly mild to moderate dermatologic reactions at array sites, and serious device‐related complications occur in fewer than 1% of cases (Mrugala et al., 2024). These findings support their compatibility with combination therapeutic approaches.

This review synthesizes current evidence on the immunomodulatory effects of TTFields, assesses their ability to enhance immunotherapy efficacy, and outlines ongoing combination strategies. It summarizes mechanisms linking TTFields to antitumor immune responses, evaluates data supporting TTFields‐immunotherapy synergy, and highlights strategies to optimize combinatorial regimens in solid tumors.

## Immunogenic Cell Death Induced by TTFields

**Figure 1:**
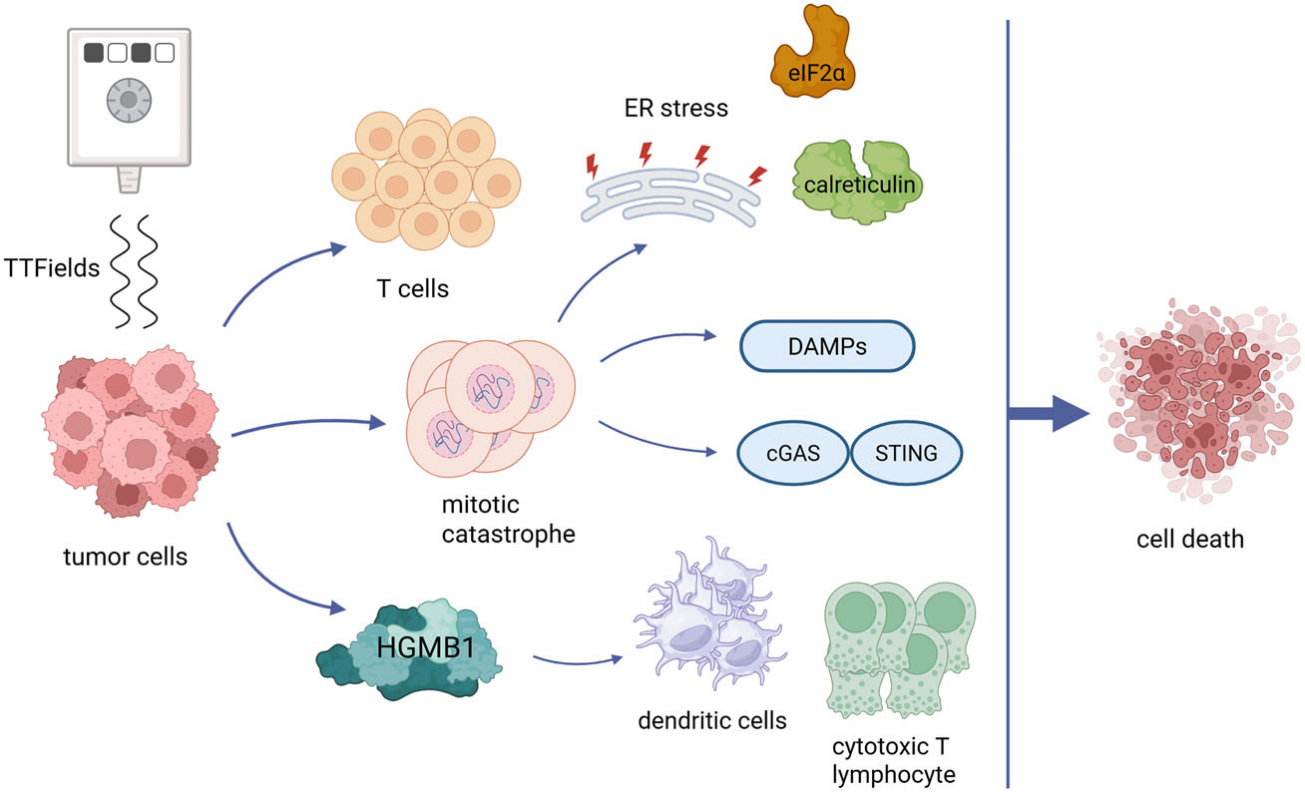
ICD induced by TTFields. TTFields disrupt mitosis in tumor cells, causing T cells proliferation, mitotic catastrophe, and release of HMGB1. These events lead to endoplasmic reticulum (ER) stress, eIF2α phosphorylation, and calreticulin exposure on the cell surface. Mitotic catastrophe also triggers the release of DAMPs and activate the cGAS–STING pathway. HMGB1 triggers DC maturation and enables CTL priming. Together, these mechanisms enhance adaptive antitumor immunity and promote ICD.

ICD is a distinct type of stress‐induced regulated cell death that triggers adaptive immune responses and supports long‐lasting immunological memory (Dou et al., 2024). Unlike non‐immunogenic apoptosis, ICD is characterized by the emission of DAMPs. Prototypical DAMPs include calreticulin, ATP, and high mobility group box 1 protein (HMGB1). Upon cellular stress or injury, these molecules activate pattern‐recognition receptors on immune and non‐immune cells to trigger host signaling pathways (Chen et al., 2025), which alert and activate innate immune cells and ultimately drive T‐cell‐mediated antitumor responses (Li et al., 2020). In recent years, TTFields have been increasingly recognized as potent inducers of ICD, thereby expanding their therapeutic relevance beyond mitotic inhibition and establishing a mechanistic bridge between biophysical tumor disruption and immune activation (summarized in Fig. 1).

More than a decade ago, Kirson et al. reported that TTFields‐treated rabbits showed significantly higher levels of CD4⁺, CD8⁺, and CD45⁺ T cells than untreated control rabbits not only in peritumoral regions but also within tumors, indicating that localized TTFields exposure can initiate systemic antitumor immune responses (Kirson et al., 2009). These findings provided conceptual evidence supporting TTFields as both a cytotoxic and an immunostimulatory therapeutic modality, challenging earlier perceptions of this approach as purely mitosis‐targeting therapy. They reveal that TTFields not only induce tumor cell death but also remodel the tumor microenvironment, fostering a more immunostimulatory and potentially therapy‐responsive state.

Mechanistically, TTFields disrupt mitosis, leading to chromosomal missegregation, prolonged mitotic arrest, and ultimately mitotic catastrophe (Giladi et al., 2015). These cellular stress events trigger endoplasmic reticulum stress, phosphorylation of the translation initiation factor eIF2α, and the surface translocation of calreticulin, a canonical “eat‐me” signal that promotes dendritic cell phagocytosis (Humeau et al., 2020). Another important dimension is the generation of abnormal aneuploid progeny as a result of mitotic catastrophe. Aneuploid cells undergo lysosomal degradation and autophagy, processes that further amplify DAMP release and increase tumor antigenicity (Stingele et al., 2012). Importantly, aneuploidy itself can act as a trigger of innate immune sensing through activation of cytosolic DNA sensors such as cyclic GMP‐AMP synthase (cGAS)‐stimulator of interferon genes (STING), thereby linking TTFields‐induced chromosomal instability to type I interferon (IFN) signaling (Basit et al., 2020). This signaling cascade represents a critical node connecting cellular stress responses to the activation of both innate and adaptive immune pathways.

At the same time, TTFields‐treated cancer cells release HMGB1, which recruits and activates dendritic cells (DCs), enhances antigen cross‐presentation, and drives dendritic cell maturation (Scaffidi et al., 2002; Yue et al., 2025; Messmer et al., 2004). TTFields have been shown to promote hallmarks of ICD that in other models are known to trigger DC maturation and antigen cross‐presentation, enabling cytotoxic T lymphocyte (CTL) priming (Moon et al., 2025). However, direct demonstration of enhanced cross‐presentation or CTL priming in TTFields‐exposed tumors remains an area for further research.

Recent in vivo studies have substantiated these mechanistic insights. In a syngeneic model of glioblastoma, Chen et al. demonstrated that TTFields‐induced immune activation achieved cure rates of 42%‐66%, which were dependent on signaling involving STING and absent in melanoma 2 (AIM2). Transcriptomic analyses of blood samples from patients with glioblastoma further revealed that TTFields activate type I IFN pathways and promote T cell clonal expansion, supporting the induction of systemic adaptive immunity (Chen et al., 2022). Collectively, these findings establish TTFields as a distinctive biophysical intervention capable of inducing ICD and activating innate immune‐sensing pathways, thereby positioning TTFields as a promising adjunct to immunotherapies, with the potential to overcome immune resistance and enhance the persistence of therapeutic responses across multiple tumor types.

## Remodeling of the Tumor Microenvironment

In addition to inducing ICD, TTFields remodel the tumor microenvironment (TME) in ways that support effective immune responses. The TME is often a major determinant of immunotherapy responsiveness, with tumors broadly classified as immune‐inflamed (“hot”), immune‐excluded, or immune‐desert (“cold”) depending on the spatial distribution and activity of immune cells (Chen & Mellman, 2017). Immune‐inflamed tumors are characterized by abundant T cell infiltration, elevated IFN‐γ signaling, increased expression of programmed death‐ligand 1 (PD‐L1), and a high tumor mutational burden. In contrast, immune‐excluded tumors often display immunosuppressive barriers that prevent T cell entry or activation, fostering immune evasion (Hegde PS et al., 2016).

**Figure 2:**
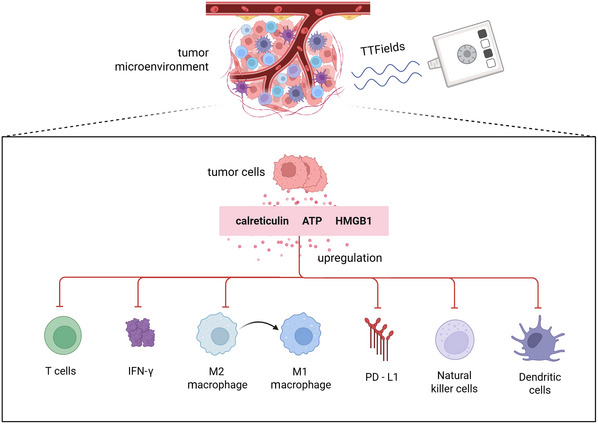
TTFields remodel the TME. TTFields promote an immune‐inflamed TME by inducing the release of calreticulin, ATP, and HMGB1 from tumor cells. These signals enhance DCs activation, drive M2‐to‐M1 macrophage polarization, increase IFN‐γ signaling and PD‐L1 expression, and boost NK and T cell activity.

TTFields have been shown to promote a shift toward a more inflamed, immunologically active TME. By triggering the release of hallmark ICD signals, including calreticulin, ATP, and HMGB1, TTFields enhance DC recruitment, maturation, and antigen cross‐presentation (Birmpilis et al., 2022; Ahmed & Tait, 2020). In parallel, TTFields influence macrophage polarization, reprogramming tumor‐associated macrophages from an immunosuppressive M2 phenotype toward a pro‐inflammatory M1 phenotype, thereby facilitating antigen presentation and T cell recruitment (Xu et al., 2023). TTFields may also enhance natural killer cell cytotoxicity by increasing the expression of stress ligands on tumor cells, rendering them more susceptible to natural‐killer‐cell‐mediated killing (Batista Napotnik et al., 2021; summarized in Fig. 2). Through these combined mechanisms, TTFields increase local immune visibility, promote CTL trafficking, and convert an immunosuppressive TME into one more permissive of immune‐mediated tumor clearance (Galon & Bruni, 2019).

In preclinical NSCLC models, TTFields increased chemokine expression (CCL2, CXCL9, CXCL10), enhanced CD4⁺ and CD8⁺ T cell infiltration, and potentiated the efficacy of immune checkpoint blockade (Lin et al., 2025; Mun et al., 2018). Similarly, in GBM models, Nitta et al. reported that concurrent therapy with TTFields and antibody to programmed cell death 1 protein (anti‐PD‐1) induced robust myeloid and T‐cell‐mediated antitumor responses, ultimately improving survival (Ryan et al., 2024).

These findings suggest that TTFields can help transform otherwise immunologically “cold” tumors such as GBM into “hot” ones more responsive to ICIs. Collectively, these findings establish TTFields as unique inducers of ICD that not only kill tumor cells directly but also remodel the immune microenvironment, laying the groundwork for durable antitumor immune memory. By enhancing immune infiltration, antigen presentation, and effector activation, TTFields provide a basis for synergistic integration with immunotherapies aimed at achieving more sustained and comprehensive tumor control.

## Combination Strategies with Immunotherapy

TTFields have been shown in preclinical studies to be highly compatible with standard‐of‐care agents that exploit DNA damage, replication stress, or mitotic checkpoint dependence, thereby enhancing therapeutic efficacy in combination settings. By increasing replicative stress, TTFields sensitize tumor cells to chemotherapies such as temozolomide, platinum agents, and gemcitabine, as well as to targeted inhibitors that rely on intact mitotic machinery (summarized in Shams & Patel, 2022; Pohling et al., 2023).

But the immunostimulatory effects of TTFields also provide a biological rationale for their combination with immunotherapies, particularly ICIs. In vivo data demonstrate that combining TTFields with anti‐PD‐1 antibodies enhances antitumor immunity and achieves greater tumor control compared to either therapy alone. TTFields‐treated cells were shown to recruit leukocytes into the peritoneal cavity; however, this recruitment was not observed at the tumor site with TTFields monotherapy. This suggests that although TTFields efficiently release DAMPs, the immunosuppressive TME may hinder the development of a fully effective antitumor immune response. Treatment with anti‐PD‐1 monotherapy produced only limited reductions in tumor volume, highlighting the added benefit of TTFields as a stimulator of immunogenic antitumor responses (Voloshin et al., 2020).

In lung tumor mouse models, TTFields combined with anti‐PD‐1, anti‐CTLA‐4 (cytotoxic T lymphocyte antigen 4), or anti‐PD‐L1 antibodies reduced tumor volume more effectively than monotherapies. This was accompanied by increased intratumoral infiltration of CTLs, higher IFN‐γ levels, and upregulation of ICD markers such as phosphorylated eIF2α and HMGB1 release, underscoring their role in enhancing checkpoint blockade efficacy (Barsheshet et al., 2022). Beyond checkpoint blockade, TTFields may complement other immune modalities by increasing antigen availability and improving the TME to support effector cell trafficking and cytotoxic function. Although direct preclinical data combining TTFields with chimeric antigen receptor T cell or tumor‐infiltrating lymphocyte therapy are limited at present, the broader literature on combination immunotherapy suggests that synergy is plausible when a treatment increases antigen presentation and T cell recruitment—effects that TTFields demonstrably produce (Grosser et al., 2019).

In a phase 2 clinical trial (NCT03405792), combining TTFields with the anti‐PD‐1 antibodies pembrolizumab and temozolomide in treating newly diagnosed glioblastoma significantly improved progression‐free (12.0 vs. 5.8 months) and overall survival (24.8 vs. 14.6 months) compared with TTFields plus temozolomide alone. TTFields activated a type‐I‐IFN‐driven immune response and promoted clonal T cell expansion, whereas pembrolizumab sustained adaptive T cell activation and memory function, highlighting the synergistic potential of TTFields and ICIs in overcoming glioblastoma's immunosuppressive microenvironment (Chen et al., 2025). Clinical evidence further supports the relevance of TME modulation. In the STELLAR trial for malignant pleural mesothelioma, TTFields combined with chemotherapy significantly prolonged survival compared with historical outcomes, highlighting their ability to improve therapeutic responses in highly immunosuppressive solid tumors. Final results from the 2‐THE‐TOP trial further substantiated these findings. The triple combination of TTFields, pembrolizumab, and maintenance temozolomide was well tolerated and yielded a median progression‐free survival (PFS) of 12.0 months versus 5.8 months and median overall survival (OS) of 24.8 months versus 14.6 months compared to matched controls treated with TTFields + TMZ. Importantly, correlative molecular analyses confirmed that TTFields monotherapy induced robust T cell activation through type I interferon pathways involving STING and AIM2 inflammasomes, thereby establishing an *in situ* vaccination effect. The subsequent addition of pembrolizumab promoted adaptive clonal switching of expanded TCRαβ clones, which strongly correlated with improved survival outcomes (Tran et al., 2023). Overall, preclinical and early clinical data strongly support TTFields as an effective partner for immunotherapy. Although challenges such as the immunosuppressive TME, patient heterogeneity, and optimal treatment sequencing remain, ongoing clinical trials aim to clarify how TTFields can be best integrated into multimodal immunotherapy regimens to maximize patient benefit (Table 1).

**Table 1:** Clinical Trials Combining TTFields with Immunotherapy

Trial	Cancer type	Combination	Phase	Outcomes
NCT03405792	Glioblastoma	TTFields + pembrolizumab + temozolomide	II	Improved PFS (12.0 vs. 5.8 months) and OS (24.8 vs. 14.6 months) compared with TTFields + temozolomide; TTFields‐induced type I IFN signaling and clonal T cell expansion (Tran et al., 2023).
NCT02397928 (STELLAR)	Malignant pleural mesothelioma	TTFields + pemetrexed + cisplatin/carboplatin	II	Median OS 18.2 months versus historical 12 months with chemotherapy alone; improved disease control in immunosuppressive TME (Ceresoli et al., 2019).
NCT02973789 (LUNAR)	NSCLC (post‐platinum, PD‐1/PD‐L1 checkpoint inhibitors allowed)	TTFields + nivolumab/pembrolizumab/atezolizumab ± chemotherapy	III	TTFields combined with systemic therapy improved OS (13.2 vs. 9.9 months); benefit consistent in patients receiving immune‐checkpoint inhibitors (Lea et al., 2023).
NCT06353360	Glioblastoma	TTFields + tislelizumab	I	Results pending.
NCT05092373	Solid tumors (abdomen or thorax)	TTFields + pembrolizumab ± chemotherapy	I	Results pending.
NCT06556563	Glioblastoma	TTFields + pembrolizumab + temozolomide	III	Results pending.
NCT05341349	Melanoma brain metastases	TTFields + pembrolizumab/nivolumab/ipilimumab	I	Results pending.

## Future Directions and Challenges

The integration of TTFields with immunotherapy offers a compelling strategy to augment antitumor immunity, yet several obstacles must be overcome to fully realize its therapeutic potential. One major challenge is the inherently immunosuppressive TME, which limits immune cell infiltration and effector function even when TTFields induce the release of immunogenic danger signals (Rousseau et al., 2025; Binnewies et al., 2018). To overcome these challenges, research is ongoing to investigate combinations of TTFields with agents that reprogram the TME—such as inhibitors of myeloid‐derived suppressor cells, regulatory T cells, or stromal remodeling therapies—to enhance immune accessibility and overcome local resistance (Lasser et al., 2024; Berg & Pietras, 2022).

Another critical need is the optimization of treatment sequencing, timing, and patient selection. The most effective strategy for integrating TTFields with chemotherapy, immunotherapy, or targeted agents remains to be determined. The identification of predictive biomarkers—such as circulating tumor DNA, DNA damage response signatures, STING pathway activation, and cytokine profiles—may enable personalized treatment planning and improve the monitoring of therapeutic response (Cristescu et al., 2018; Kim et al., 2016).

Expanding the clinical application of TTFields to additional solid tumors, while ensuring patient compliance and minimizing device‐related toxicities, is also a priority. Technological advances—including tumor‐type‐specific optimization of field frequencies and improved delivery systems—could enhance efficacy and broaden accessibility. In addition, comprehensive correlative analyses from ongoing clinical trials will be essential to clarify the immunologic correlates of response and resistance, guiding the rational design of next‐generation combination regimens. In parallel, careful consideration of regulatory, logistical, and economic factors will be essential to support widespread clinical implementation.

Overall, these efforts will be critical to realizing the full therapeutic potential of TTFields, particularly in combination with immunotherapy, and translating their preclinical promise into durable and clinically meaningful benefits for patients across diverse malignancies.

## Conclusion

TTFields represent a non‐invasive therapeutic modality that not only inhibits tumor proliferation but also activates systemic antitumor immunity through the induction of ICD and remodeling of the TME. Preclinical and early clinical evidence demonstrates that TTFields synergize with ICIs, promoting enhanced T cell infiltration, activation, and memory formation, even in immunologically “cold” tumors. By integrating direct cytotoxic effects with immune modulation, TTFields provide a compelling strategy to augment current immunotherapeutic approaches. Moreover, their unique biophysical mechanism allows broad compatibility with various therapeutic platforms, minimizing overlapping toxicities and enabling rational combination designs. As mechanistic insights continue to expand, TTFields may ultimately serve as a cornerstone in multimodal regimens aimed at converting immune‐resistant tumors into durable responders. Future research should prioritize optimizing combination regimens, elucidating underlying molecular and immunological mechanisms, and identifying predictive biomarkers to maximize clinical efficacy and broaden the therapeutic reach of TTFields across diverse tumor types.

## Author Contributions

**Menglan Liu**: Conceptualization; investigation; writing—original draft; writing—review and editing. **Maria El Hage**: Validation; writing—review and editing. **Jihui Zhao**: Formal analysis; visualization. **Michael Linnebacher**: Conceptualization; supervision; writing—original draft; writing—review and editing.

## Conflict of Interest

The authors declare no conflict of interest.

## Data Availability

Data sharing not applicable ‐ no new data generated, or the article describes entirely theoretical research.
